# A low cost surrogate eye model for corneal foreign body removal

**DOI:** 10.1186/s12886-020-1310-z

**Published:** 2020-02-07

**Authors:** Jingping Lin, Mui Teng Chua

**Affiliations:** 1 Emergency Medicine Department, National University Hospital, Level 4, National University Centre for Oral Health, 9 Lower Kent Ridge Road, Singapore, 119085 Singapore; 2 Department of Surgery, Yong Loo Lin School of Medicine, National University of Singapore, Singapore, Singapore

**Keywords:** Simulation, Medical education, Emergency medicine, Ophthalmology, Curriculum, Cornea, Foreign body, Intraocular

## Abstract

**Background:**

Patients commonly present to the Emergency Department with a corneal foreign body (FB). There is currently a lack of adequate training for junior doctors in the management of this condition. Our self-made surrogate eye model aims to address this void in our junior doctors’ knowledge.

**Methods:**

Participants were guided through a hands-on session with a slit-lamp using our eye model, which is made of a hemispherical agar embedded with pencil lead fragments simulating as FBs. Using a 7-point Likert scale, all participants completed a questionnaire both before and after training, for: (1) knowledge in corneal FB removal, (2) confidence in corneal FB removal, and (3) effectiveness of the model.

**Results:**

Out of 73 participants, 82.2% (60/73) had no prior experience in corneal FBs removal. After the training session, their knowledge improved from a median score of 2 (interquartile range [IQR] 1 to 3) to 5 (IQR 5 to 6), with improvement in confidence levels from 2 (IQR 1 to 2) to 5 (IQR 4 to 6). The effectiveness of our eye model scored a median of 6 (IQR 5 to 7).

**Conclusions:**

Our surrogate eye model is low-cost, quick and easy to reproduce. After use, our learners expressed greater confidence in managing the removal of corneal FBs and use of slit lamp. With a recent focus in patient safety and quality, teaching this procedure via simulation is a safe way of bridging the gap between traditional didactic teaching and the clinical environment.

## Background

Patients with a corneal foreign body (FB) form an important group of people presenting to the Emergency Department (ED) with ocular complaints [1, 2]. This makes it important for doctors working in the ED to be familiar with the procedure of corneal FB removal. ED staffing with junior doctors is a common occurrence worldwide [3], resulting in this group of doctors seeing many of these patients. Yet, most of them have little to no experience in dealing with corneal FBs. Throughout the past decades, approximately 70% of junior doctors have a lack of confidence in dealing with eye emergencies, both locally and internationally [4, 5]. Additionally, undergraduate training in ophthalmological conditions and emergencies are inadequate and widely variable, further compounding this problem [6].

It is imperative for junior ED doctors to be proficient in the evaluation and management of corneal FBs since errors can potentially cause adverse sight-threatening complications such as corneal perforation [7–9]. However, the removal of corneal FBs is not an ideal procedure to teach on an actual patient, as it can be intimidating for both the patient and doctor. We were unable to find commercially available eye models for educational purposes, so we created a safe and effective model to teach the procedure of corneal FBs removal.

Sited in a tertiary academic institution, our ED has an annual census of approximately 110,000 patients, ranging from Patient Acuity Category Status (PACS) of 1 to 3, where a patient with PACS 1 status requires immediate medical attention and a patient with PACS 3 status is ambulant, with mild to moderate acute symptoms [10]. Among the attendances, the number of patients presenting with corneal FBs averages about 250 per year. Our ED is primarily staffed by junior doctors who are at least postgraduate year (PGY) 2 and above. On-site supervision by board-certified emergency physicians are available round-the-clock, and a fully equipped consult room with a slit-lamp is dedicated for use on patients with ophthalmological complaints.

There are three medical schools in Singapore, two of which provide undergraduate medical education whilst the other provides postgraduate studies. Of the 3 medical schools, the National University of Singapore (NUS) Yong Loo Lin School of Medicine produces the most number of graduates who join the public hospitals yearly [11]. The ophthalmology curriculum in NUS only includes an hour-long didactic lecture, bedside tutorials, and operating theatre observation. There is a practical demonstration to introduce students to skills such as slit-lamp examination. However, there are no hands-on sessions in the curriculum for training of corneal FB removal [12].

Here, we describe our method of creating a cheap and easy eye model that can be used for training corneal FB removal. The primary objective of this study was to evaluate the effectiveness of this eye model in improving the knowledge and confidence of our junior doctors in the removal of corneal FBs.

## Methods

We conducted a prospective study between April and November 2016, using our novel, low-cost eye model, among postgraduate learners in the ED of a Singapore hospital. Our hospital is a tertiary academic medical institution with a structured Emergency Medicine residency-training program accredited by the Accreditation Council for Graduate Medical Education International. Ethics approval for waiver of consent was obtained from the National University of Singapore’s (NUS) Institutional Review Board (NUS-IRB reference code: NUS-IRB Review B-16-058).

### Model construction

**Fig. 1:**
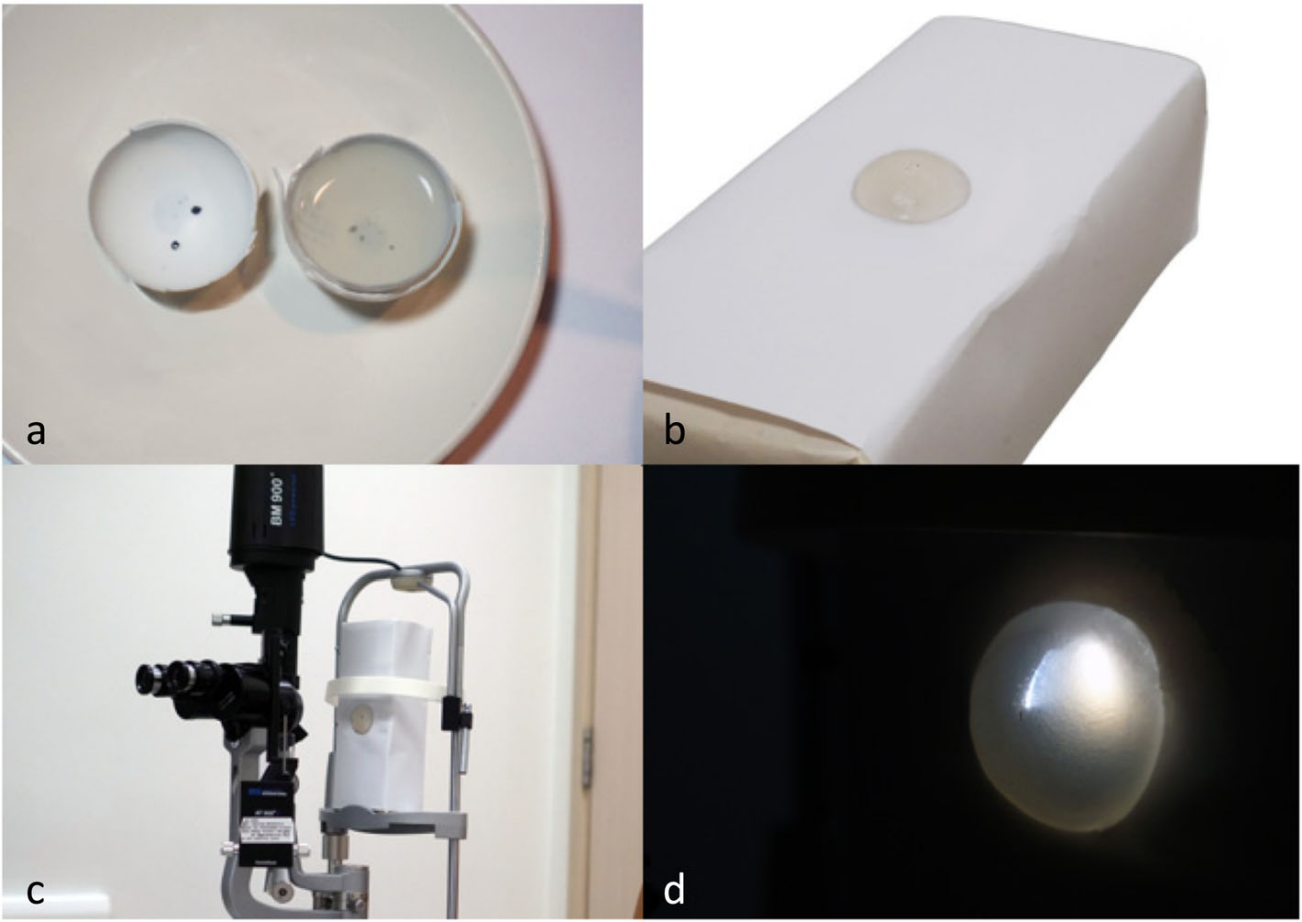
Construction of the eye model. **a**: A table tennis ball was halved and used as a mould. Pencil lead fragments added to the mould and agar solution added into the mould. **b**: Completed eye model mounted onto box with glue. **c**: Eye model mounted on a slit lamp. **d**: A view of the eye model with a slit lamp beam cast upon it


To make our model (see Additional file 1: Video S1), the following materials were used: (1) agar powder, (2) water, (3) table tennis ball and (4) pencil lead shavings (Table 1). First, the table tennis ball was cut into half to simulate the hemispherical shape of the eye. Next, pencil lead shavings were scattered into the base of the moulds to simulate corneal FBs. After mixing 3 g of agar powder with 230mls of water, the water was brought to a boil and left until the agar powder completely dissolved. This solution was then poured into the pre-prepared moulds and refrigerated until firm, as seen in Fig. 1a. If the lead shavings floated upwards after pouring in the solution, they were pushed to the bottom with a pair of tweezers to ensure correct positioning. Excluding the time required to refrigerate, the entire process can be completed within 5 min. Once firm, the agar model was unmoulded. These were made in large batches and stored in a fridge until ready for use. Just before each session, the model was glued onto a box (Fig. 1b), which was then secured with tape onto the chin rest of the slit lamp as seen in Fig. 1c. Figure 1d shows the eye model when the slit lamp beam is cast upon it.

**Table 1:** Ingredients and costs for the eye model construction

Materials	Quantity	Cost (Singapore Dollars)	Cost (US Dollars)
Agar powder	3 g	1.10	0.82
Table tennis ball	1, halved	0.10	0.08
Pencil lead shavings	1 g	0.10	0.08
Total cost		1.30	0.98

**Additional file 1:** Video of eye model construction..


### Training structure

Junior doctors were divided into groups of 3 and assigned to an ED senior resident for a 2-h hands-on session. The ED senior resident conducting the training sessions would have completed the intermediate exams in Emergency Medicine (either the Membership of the Royal College of Emergency Medicine [United Kingdom]) or the Master of Medicine in Emergency Medicine [Singapore]) and completed at least 2 weeks of clinical rotation in Ophthalmology.

The training curriculum comprised of 2 parts. Every participant was required to complete a set of pre-reading materials prior to attending the tutorial. Basic slit-lamp use was then taught together with the technique of FBs removal using a 27-gauge needle. A standard teaching template was provided for each educator’s adherence to ensure every session’s homogeneity. During the teaching session, a set of pre-prepared slides was also used to augment the learning experience.

### Pre- and post- training evaluation

The junior doctors completed a self-assessed questionnaire (Table 2) before and after the session for confidence and knowledge in corneal FBs removal, and in effectiveness of the eye model. The questionnaire was scored on a 7-point Likert scale.

**Table 2:** Questionnaire done both pre- and post- training

1. I have very good knowledge of slit lamp usage*	
2. I have very good knowledge of corneal FBs removal*	
3. I am very confident of using the slit-lamp for anterior segment evaluation*	
4. I am very confident of assessing depth of FBs*	
5. I am very confident of removing corneal FBs*	

*Assessed using a 7-point Likert scale where 1 is “strongly disagree”, 4 is “somewhat agree”, and 7 is “strongly agree””

### Statistical analysis

Results were analyzed using Stata 14 (StataCorp LP, College Station, TX). Proportions were expressed in percentages and non-parametric variables were analyzed using the Mann Whitney U test.

## Results

### Demographics

A total of 73 junior doctors participated in this study between April and November 2016. Their median age was 26 years old (interquartile range [IQR] 25 to 29 years old), with an overall median PGY of training at 2 years (IQR 2 to 4 years). Table 3 outlines the demographics of all our participants.

**Table 3:** Demographics of participants

Characteristic	Number, n (*n* = 73)	%
Gender		
Male	47	64.4
Female	26	35.6
Age		
20–25	26	35.6
26–30	37	50.7
31–35	7	9.6
36–40	3	4.1
Training status		
Non-trainees	57	78.1
Trainee	16	21.9
Previous ophthalmology posting		
Yes	5	6.8
No	66	90.4
Missing data	2	2.7
Medical School		
Singapore	32	43.8
Overseas	41	56.2
Experience in corneal FBs removal		
Had prior experience	13	17.8
No experience	60	82.2

### Confidence and knowledge

**Fig. 2:**
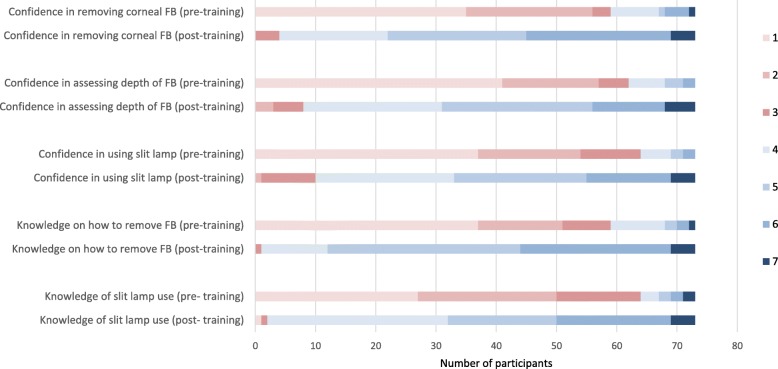
Participants’ questionnaire responses using the 7-point Likert Scale

Prior to the training session, both the participants’ knowledge in slit-lamp use and corneal FBs removal scored a median of 2 (IQR 1 to 3). After training, both indicators improved to a median score of 4.5 (IQR 4 to 5.5) and 5 (IQR 5 to 6) respectively. Their confidence in slit-lamp use and corneal FBs removal improved from a median of 1 (IQR 1 to 2.5) and 2 (IQR 1 to 2) to a median of 5 (IQR 4 to 5) and 5 (IQR 4 to 6), respectively (all *p* values < 0.001). Their confidence in assessing the depth of corneal FBs also improved significantly from 1 (IQR 1 to 2) pre-training to 5 (IQR 4 to 5, *p* < 0.001) post-training. Figure 2 illustrates our participants’ questionnaire responses.

### Effectiveness of eye model

Using the eye model, the ease of visualization of corneal FB was excellent with a median score of 6 (IQR 5 to 7). The study participants also felt that the model was very effective in teaching the skill of removing corneal FB (median score of 6, IQR 5 to 6) and very realistic in simulating a corneal FB (median score of 5, IQR 4 to 6). Overall, the effectiveness of the eye model in teaching FB removal scored a median of 6 (IQR 5 to 7).

**Fig. 3:**
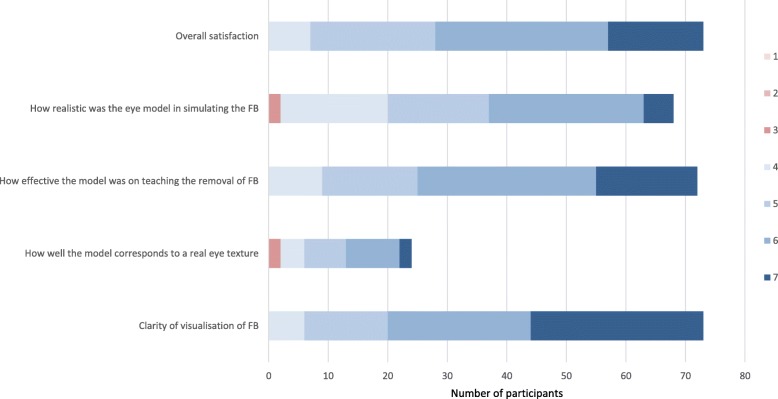
Participants’ responses on the effectiveness of the eye model

Among the participants who had prior experience in removing corneal FBs, they felt that the eye model corresponded relatively well to the real eye in terms of texture (median score 5, IQR 4.5 to 6). Figure 3 illustrates the overall impressions of our participants on our eye model. Participants were asked to omit answering how well the model corresponds to a real eye texture or in simulating FBs if they have not had any prior experience in removing corneal FBs.

## Discussion

With the use of simple, low-cost and easily available materials, we successfully constructed an eye model that is effective in improving junior doctors’ confidence in removing corneal FBs. It has widespread acceptance amongst the study participants in terms of realism and resemblance to real corneal texture.

There have been several described attempts at creating a simulation model to teach this procedure. In 1995, Austin et al. described the use of glass spheres with a film of paraffin, with embedded pieces of metal to simulate corneal FBs. Participants reported improved comfort and skill in removing them [13]. In 2015, Cheng et al. developed a model using unused but expired microbiology agar plates with gravel simulating corneal FBs [14].

In 2016, Gallagher et al. compared different simulation models, using a variety of different materials. In their first model, they used a cardboard box with ink simulating the corneal FBs. In their second model, polyvinyl resin and gelatin were used to create the eyeball, and ground pepper was used to simulate corneal FBs. Their third model made use of a glass sphere and wax to simulate the eyeball, and melted crayon was used as the corneal FB. Their final model was an agar plate simulating the eye, with peppercorn as corneal FBs [1]. More recently in 2017, a task-trainer using ballistics gel, silicone, paint thinner, baby oil, petroleum jelly and cornstarch was created for under USD$75. This task-trainer also included the use of a Styrofoam mannequin head and reported a time of approximately 90 min to complete [15]. There have even been literature describing the use of bovine eyes as a way to teach this procedure [16].

It is thus evident that teaching the removal of corneal FBs is a procedure that can be taught via simulation. Having looked at all the available literature at making a simulation eye model, we feel that our low-cost, low-tech model has an advantage over the others, for the following reasons: [1] our model approximates the consistency of a human cornea well, [2] its hemispherical shape simulates that of the eye ball and allows users to appreciate depth of the corneal FBs, and [3] it is quick, easy, and cheap (under USD$1) to re-create and particularly suited for an environment with tight resources.

We have established that the majority of our junior doctors lack experience in dealing with eye emergencies. This problem is not unique to us [4, 5]. Before the commencement of this training curriculum in 2016, teaching of corneal FBs removal to junior doctors was largely opportunistic in our institution. On a regular shift, junior doctors manage patients independently and would consult a senior doctor if they encounter problems. If the senior doctor was occupied with other patients, they may not have enough time to teach the procedure and these patients are then referred to the on-call ophthalmology service for removal of the FBs. Learning opportunity lost aside, this potentially lengthens the throughput time of the patient in the ED, contributing to the perennial issues of overcrowding. Increasingly, we are also seeing patients who understandably, do not want to be “practiced” upon, and frequently ask for an experienced physician to perform the procedure for him or her, making it even more difficult to teach a young doctor this crucial procedure.

Using simulation in medical education is now widespread and this phenomenon is in part, contributed by changes in patients’ healthcare expectations, as well as changes in our academic environment, with renewed emphasis on patient safety, making it more difficult to “practice” directly on an actual patient [17–20]. The use of simulation bypasses many of the issues that arise from the traditional “see one, do one, teach one” method: they are readily available at any time, and allow trainees to practice their skills in an environment free of risk [13, 21].

Patient safety is an important element in our daily practice as emergency physicians. This ties in closely with important ethical issues about the appropriateness of “using” actual patients as training resources. Teaching the removal of corneal FBs using our surrogate eye model circumvents such ethical issues and improves patient safety. It is safer for patients and allows doctors to be confident with their skills before performing the procedure on an actual patient.

Apart from teaching, educators can also use simulation as a means of evaluating competency in a procedure [22]. Immediate feedback and guidance can also be given during each session without any threat to patient safety [13]. The educational experience is now learner-centered, instead of being patient-centered, as would be appropriate in real-live clinical situations [21]. Simulation based teaching has been shown to be more effective than didactic teaching alone for various subjects [23] and we strongly believe in the effectiveness of our model.

Using a simulation model provides useful practice of a procedural skill requiring eye-hand coordination prior to application of these skills in real-life clinical practice.

### Limitations

We acknowledge that the fidelity of simulation training is never completely identical to the real-life patient and that there is no validation on how well agar compares to cornea. For example, it is difficult to simulate a perforated globe using our agar model if the participant makes an error during the removal of the corneal FBs. The real eye is also mobile and the element of blinking in a real patient is difficult to simulate in our model. We have tried to circumvent this problem by showing participants videos during the session and explaining how this would appear under the slit lamp using our simulated model, which we felt was much better appreciated than just reading on their own. Additionally, we have an instructor present to guide and correct each learner, making the teaching individualized and targeted at resolving each learner’s doubts. Given how cheap and readily available agar is, we believe this is still a good initial training tool to provide to junior clinicians.

The transparency of our model can be improved, as it is sometimes difficult to assess the depth of the FBs if the particular model used has a cloudier consistency. Additionally, our model is not re-useable, although it can easily be reproduced at a low cost.

With these issues in mind, we have now developed a re-usable model using a 3-dimensional computer design to print a human face with eye sockets that can accommodate artificial globes made of gelatin, glycerine and water. This model has thus far been promising in simulating the eye, with an improvement in simulating the transparency of the cornea. However, we feel it is still relevant and important to present our agar model because this new re-usable model may not be as readily available to some as, similar to previously described models in literature, it requires more expertise and specialized equipment to manufacture.

There were limitations in our study methodology. Participants were evaluated with a self-administered questionnaire without assessment of objective outcomes such as size of corneal defect after FB removal and these results may not translate to clinical competency in real-life scenarios. Our methodology mainly assessed our learners’ reaction to the training and was unable to completely assess task performance or knowledge objectively. We were also not able to ascertain if using just video training with live teaching and slit lamp training would have resulted in the same improvements without hands-on teaching with our eye model, as this was not part of our study design.

To adequately ascertain clinical competency, the learners will have to be assessed while performing the procedure on a live patient with corneal FBs, which would be opportunistic and may not be ethical. As the assessment collected has been de-identified in accordance to our Institutional Review Board’s regulations for consent exemption, we were also unable to trace the individual’s ability to perform this procedure on real patients after the training.

Moving forward, we intend to use this study as a proof of concept and will collect data using experienced clinicians (senior emergency physicians or ophthalmologists) to evaluate the fidelity and suitability of our model as a surrogate.

## Conclusion

We have described an innovative, affordable and easily reproducible method of creating a surrogate eye model. Our model has proven to be sustainable and an acceptable teaching method among junior emergency doctors. Learners expressed greater confidence in managing removal of corneal FBs and use of slit lamp. Given the easy and inexpensive way in which it was constructed, we feel strongly that our model has huge potential applications, especially in training settings where resources are limited.

## Data Availability

All data generated or analyzed during this study are included in this published article.

## Abbreviations

ED: Emergency Department
FB: Foreign body
NUS: National University of Singapore
PACS: Patient acuity category status
